# Circular analysis in complex stochastic systems

**DOI:** 10.1038/srep17986

**Published:** 2015-12-10

**Authors:** Angelo Valleriani

**Affiliations:** 1 Max Planck Institute of Colloids and Interfaces, Department of Theory and Bio-Systems, Potsdam, 14424, Germany

## Abstract

Ruling out observations can lead to wrong models. This danger occurs unwillingly when one selects observations, experiments, simulations or time-series based on their outcome. In stochastic processes, conditioning on the future outcome biases all local transition probabilities and makes them consistent with the selected outcome. This circular self-consistency leads to models that are inconsistent with physical reality. It is also the reason why models built solely on macroscopic observations are prone to this fallacy.

Observations are at the core of scientific discovery. When observations come in the form of time series, they deliver a record of past events and allow us to build models to understand the necessary relationships between events^1^,^2^,^3^,^4^. Since the present does not affect the past, we would believe that a model derived from a time series of past events is not influenced by the future outcome of the entire process. However, when a subset of all the possible time series is selected based on its final outcome, a circular argument is at work.

Circular analysis^5^ has long been discussed as a possibly misleading procedure in experimental science, especially in neuroscience. Known also as “double-dipping”, it generically consists of preselecting a subset of all the possible events for further theoretical or statistical analysis, thus obtaining a consistency and statistical significance beyond the real nature of the phenomenon. So far, the analysis of circularity has been proposed in terms of negative examples, where it is shown how “double-dipping” leads to artificially highly significant results^5^. However, a more general theoretical understanding of the effects of “double-dipping” is still missing.

The effects of “double-dipping” are particularly relevant in building models from huge collections of data, as it is common for instance in systems biology^4^ and other complex systems^6^,^7^. Known as reverse engineering, in its extreme formulation this procedure aims to infer yet unknown causal relationships between variables solely from data analysis. Despite the inherent difficulty of finding a unique solution to such inverse problem, the resulting models (probabilistic and/or deterministic) often show a very good level of consistency with the data. Yet, loud criticisms to this procedure condemn it to fail completely^8^,^9^ because the inverse problem has too many solutions and the data is too noisy. Nevertheless, a serious point is that data are often collected under the (sometimes implicit) condition that a certain outcome happens (e.g. the cells did not die during the experiment, a target metabolite shows up in sufficiently high concentration, the amount of transcripts is large enough, the polysomes look reasonable etc.). As we shall see, selecting the data based on outcomes does not spoil the possibility to build models consistent with the macroscopic observations. Still, those models are likely to be inconsistent with the underlying microscopic mechanisms.

Two rather popular books^10^,^11^ have made it clear how selection of subsets of events based on their outcome is also a common fallacy in everyday life. Every time we judge the success probability of a certain choice based only on our personal experience, we commit a mistake unless we have either a clear understanding of the true mechanisms of success or enough statistics beyond our personal experience^12^. Similarly, this phenomenon occurs whenever one tries to extract the “rules of success” from a set of particularly successful life histories. Indeed, life histories are like random trajectories and the selection of the successful ones is like selecting them based on their final outcome (*i.e.*, success). As we shall see, the drunkard’s walk teaches us that we get very biased lessons from them. Technically, the selection of the trajectories based on their outcome corresponds to conditioning the process in its future. This conditioning introduces a bias known as the Doob’s *h*-transform in the mathematical literature on stochastic processes and in various contexts from physics, chemistry, and biology^13^,^14^,^15^,^16^,^17^,^18^. The purpose of this work is to clarify, by means of a simple model, the relationship between the effect of “double-dipping” and the Doob’s *h*-transform. This attempt is built on current knowledge on conditioned stochastic processes and in particular on conditioned Markov chains. This should contribute to establish a link between conditioned processes and the difficulty to gain knowledge from macroscopic observations alone.

## Results

**Figure 1:**
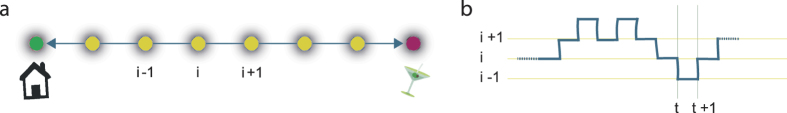
The drunkard’s walk. (**a**) At each position *i* between home and the bar the drunkard can step either to the right (position *i* + 1) or to the left (position *i* − 1) with equal probability *p* = *q* = 0.5. When the drunkard reaches either home (green dot) or the bar (purple dot) the walk terminates. A new walk starts at a random position in one of the intermediate (yellow) dots. **(b)** A small piece of a random trajectory shows that at step *t* the walker jumps from position *i* to *i* − 1 and at step *t* + 1 the walker jumps from *i* − 1 to position *i*. A large collection of statistically independent trajectories provides the basis for an empirical estimation of the jump probabilities.

Simple, familiar models help us sharpen our skill to detect subtle effects in more complex, real world situations. Consider, to this purpose, the drunkard’s walk. The drunkard jumps randomly either left or right with equal probabilities until it goes home or it enters the bar (Fig. 1a). A large collection of independent trajectories (Fig. 1b) could provide the empirical basis to compute the jump probabilities if these were unknown.

**Figure 2:**
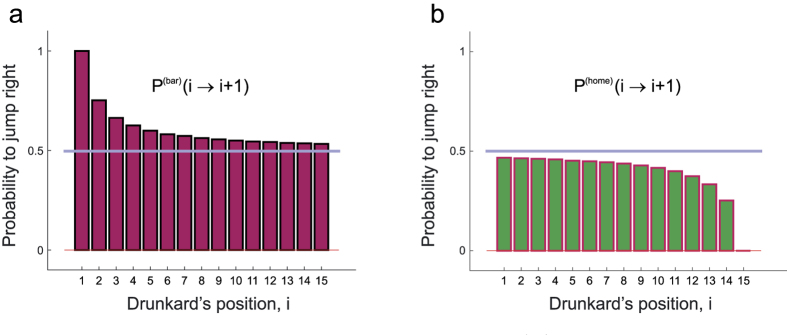
Jump probabilities. (**a**) The probabilities to jump right, 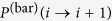 computed from a collection of trajectories ending at the bar (position 16 in this plot) appear to be systematically larger than 0.5 (horizontal line). For instance, when the walker is at position *i* = 2, it seems to jump to position *i* = 3 three times out of every four visits. In reality, according to the local mechanisms used to generate the walk the jump probability is 0.5 at each position. (**b**) When the same analysis is performed from a collection of trajectories ending at home, the probabilities 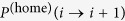 to jump right are systematically smaller than 0.5.

As already mentioned, the drunkard will eventually reach either home or the bar. Let us now consider the point of view of an observer who retains only the subset of independent trajectories ending at the bar. The observer wants to compute the jumping probabilities from any position *i* to its neighboring position *i* + 1 out of this subset of trajectories. To this purpose, the observer counts the observed transitions from *i* to *i* + 1 and uses the resulting number, 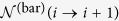, to compute the jumping probability as


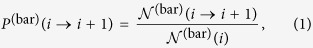


where 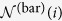 is the total number of observed visits in position *i* in the subset of trajectories ending at the bar. One can compute 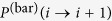 analytically (Supplementary note) or compute it numerically. In the last case, we assume here that we always have as many trajectories as necessary to consider Eq. (1) sufficiently accurate for any purpose^19^. It turns out that the probability to jump towards the bar is systematically larger than 0.5 and depends on the position of the walker (Fig 2a), contrary to the local rules used to generate the drunkard’s walk. Had the observer chosen to analyze the trajectories ending at home, instead, he would have found a somewhat specular result for the same quantity 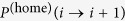 (Fig. 2b). Thus, the selection of the trajectories based on the final outcome has introduced a bias in *all* jump probabilities of the walk so that their estimated values are not the true, physical ones.

The astonishing part of this result comes if one considers the following scenario. Imagine if the observer had access only to the trajectories leading to the bar while it had really no access to the “home” trajectories, completely ignores that they could exist at all, and obtains the result of the statistical analysis (Fig. 2a): What kind of conclusion about the physics behind this result would the observer come to? In the absence of any other information, the observer would have to conclude that there is a *local* drift towards right where there is none in reality. The jump probabilities derived in this way would make perfectly sense, there would be no way to invalidate the result but the model they seem to support is wrong.

### Derivation

**Figure 3:**
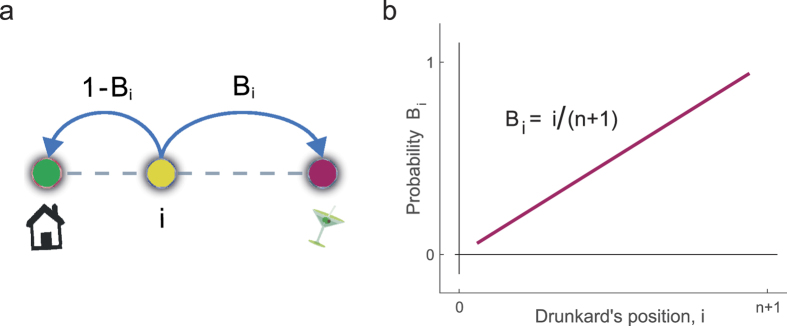
Probability to end-up at the bar. (**a**) When the drunkard is at any position *i* it will eventually reach home or the bar. The probability 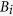 to reach the bar will depend on *i* and on the values of the transition probabilities at each position. From position *i* the drunkard will reach home with probability 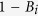. **(b)** Home is positioned at 0 and the bar is placed at position *n* + 1, where *n* is the number of intermediate positions. For 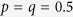 the probability 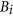 to reach the bar before home grows linearly with the drunkard’s position *i*. For other networks and other choices of the transition probabilities the linear relationship may get lost but the probability can be still computed analytically using algebraic methods (Supplementary note).

On the technical side, let us first define the conditional probability 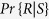 that event 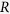 occurs given that event *S* holds true. This conditional probability is obtained from the joint probability 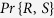 that both events *R* and *S* occur, through 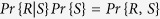. Now, let 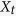 denote the random variable that gives the position of the drunkard after step *t* and let 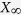 denote the final position of the drunkard at the end of each walk, namely either “home” or “bar”. Thus, the probability expressed in Eq. (1) is explicitly defined as


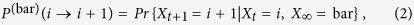


where the second condition in the right hand side is the one that selects the trajectories ending at the bar. By applying the definition of conditional probability to Eq. (2), in addition to the memoryless property of the random walk, lead to the following mathematical relationship between 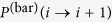 and 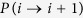



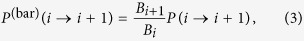


where 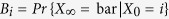 is the probability to end-up at the bar when the walk is at position *i* (Fig 3a), and 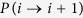 is the true jump probability. The relationship given in Eq. (3) is the Doob’s *h*-transform mentioned earlier. Its analytical expression for the drunkard’s walk (Fig. 3b) leads to


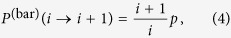


where *p* = 0.5 (Supplementary note).

### Generalizations

**Figure 4:**
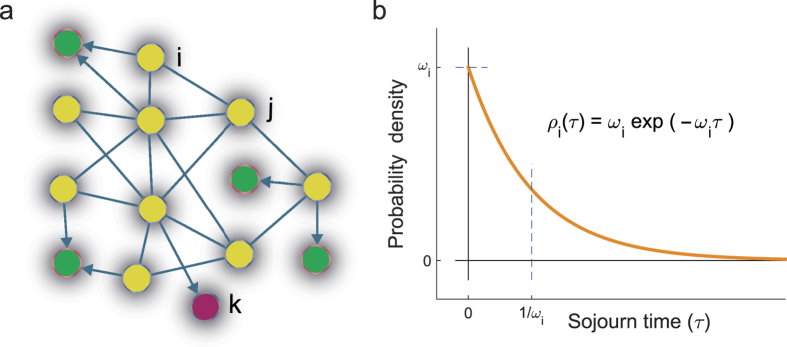
Network of states. (**a**) In a generic network of states with two or more outcomes, we might consider only the subset of trajectories ending in state *k*. On this set of trajectories, the conditioned transition rate 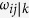 is different from the unconditioned (true) transition rate 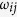. (**b**) In a continuous time Markov chain the sojourn time on each transient state *i* is exponentially distributed, with a parameter 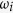 given by the sum of the rates outgoing from *i*. Application of the Doob’s *h*-transform reshapes the single rates but their sum remains constant so that the sojourn time on each state does not depend on the conditioning on the future outcome.

In arbitrarily complex networks of states the selection of the trajectories leads to a shift formally similar to Eq. (3) both of the rates (in continuous time) and of the probabilities. Consider indeed an abstract network of states of a Markov chain in continuous time (Figs. 4a-4b). The set of states is characterized by several transient states (in yellow) and more than one absorbing states (green and purple). Let *X*(*t*) be the random variable giving the state of the process at time *t* (in this section *t* is continuous). The transitions of this variable over the set of states are defined through the infinitesimal conditional probabilities


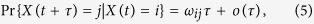


for infinitesimal 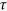, where 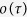 are terms of order higher than 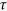. The rates 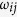 are the physical quantities that govern these transitions. In physical or biochemical systems, indeed, they are often expressed in terms of fundamental quantities such as free energy differences. The rates, thus, are the quantities that contain the microscopic physical reality of the system. It would seem therefore counterintuitive that when measured from an entire time series the values of the rates may depend on the last measurement at the end of the time series. However, this is what happens. Consider indeed the set of realizations of the process ending at the absorbing state *k* (Fig. 4a). The transition probabilities are now conditioned so that *k* is the outcome of the process. These probabilities are governed by the conditional transition rates 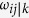



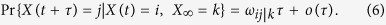


If 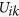 denotes the absorption probability in *k* starting from *i*, using Bayes rules on Eq. (6), the following relationship


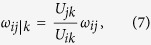


holds for all *i* and *j* (Supplementary note). A similar relationship, based on the same arguments but in a different context, was derived also earlier^13^,^15^,^18^. Since in general the absorption probabilities in Eq. (7) are different, *i.e.*, 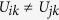, this equation implies that measurements performed under a condition based on the outcome of the process (here 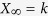) are likely to lead to biased transition rates 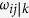 and thus to a wrong estimate of the physical quantities governing the process. A relationship of this kind holds also when instead of conditioning in just one state, the trajectories are conditioned on a subset of absorbing states. A similar effect is present also when the system is in steady state and the final state can be visited several times before the trajectory is stopped (Supplementary note), when realizations of an equilibrium process are selected to satisfy certain non-equilibrium conditions^14^ or when a process is conditioned on rare events to happen^16^.

The following example may help to understand the relevance of this result. Consider a stochastic process that involves three states, *A*, *T*, and *B* connected in a linear reversible chain as


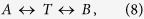


where the transition rates are 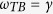 and 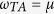 (the other rates have to be non zero but are not relevant for what follows). This system obeys detailed balance for any choice of the rates, whenever they are all different from zero. Suppose now that we decide to find the estimate 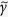 of the true rate 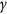 according to the following procedure. We consider a set of trajectories that always start in *A* and reach *B* after visiting the transition state *T* without returning back to *A*. Therefore, all trajectories of the kind 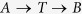 are kept and all those that start with 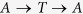 are discarded. This is equivalent to consider trajectories conditioned to start in *A* and to reach *B* before reaching *A* again. Applying Eq. (7) leads to


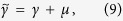


which means that the estimated rate is always larger than the true one. If *A* and *B* are two physical states or configurations, the estimated rate 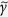 will always make *B* appear more stable than *A* even when the contrary holds.

## Discussion

Reverse engineering, a technique often used in systems biology to infer models from sets of data^4^,^6^,^7^, is particularly sensitive to the sort of bias described here. It has already been recognized, though in a rather qualitative fashion, that the program of deriving models solely from data often corresponds to an ill-defined inverse problem^8^,^9^. One suggested solution is to construct as many physically meaningful models as possible and then use the data to falsify as many of them as possible^8^. Within the metaphor in which only the drunkard’s trajectories leading to the bar are available, only a model with drift would make perfectly sense and would survive the falsification test. This model would be able to make predictions consistent with the observed data but it would still be wrong. Another suggestion is to exploit the reductionist approach, *i.e.*, construct a microscopic theory of the background processes and to observe those microscopic events to finally build a comprehensive theory^9^. In the metaphor of our drunkard’s walk this corresponds to place, at each position *i*, an observer who counts the transitions to neighboring positions regardless of what is the final outcome. The observer would come to the conclusion that the physics behind the process is a coin toss. This would reveal a contradiction with the jump probabilities computed from solving the inverse problem and thus correctly discard the model with drift. This approach would certainly be an advancement. Still, it shifts the issue one level below. Indeed, also the jumping process from *i* to *i* + 1 could involve a complex set of deeper microscopic processes for which the jump is the corresponding macroscopic event. Since there is no clear limit to how much microscopic one should go, this is an ever ending process.

In physical systems, it has already been recognized that conditioning in the future leads to different physical laws compared to the unconditioned process^14^,^16^,^20^. This happens when observations or trajectories are analyzed after a selection based on their outcome. This may occur when computer simulations of natural phenomena are prematurely stopped and discarded if they do not reach a certain target^21^,^22^,^23^. As an example, consider the case when computer simulations aimed at exploring transition rates are discarded because they do not reach the desired conformations or coordinates. Discarding those simulations is equivalent to selecting the trajectories based on their outcome and can be described as a process conditioned on a pre-selected absorbing state (Fig. 4a). The computation of the transition rates from those trajectories is then affected by the Doob’s *h*-transform and the computed rates of local transitions do not correspond to the physical ones. In analogy, this selection occurs when experiments that “did not work” or negative results are discarded or ignored^24^,^25^.

Also rather natural assumptions made in modeling stochastic phenomena may hide a selection with a potential bias. An example concerns modeling of molecular motors such as kinesin. Kinesin is a biological nanomachine that performs a complex driven random walk on a filament and tends to detach from the filament due to thermal fluctuations. However, often kinesin is modeled as if it would never detach from the filament^15^,^26^,^27^. Practically, this means that one considers only the trajectories in which the detachment time is longer than the run time. This is a kind of conditioning in the future behavior of the process. When the experimental trajectories are eventually used to determine the rates that govern the transitions between the motor’s internal states, the Doob’s *h*-transform enters into play and the rates thus derived are not the true rates of the process, unless one takes the effect of the bias properly into account^28^.

Even more significant is this effect when only one long trajectory (one realization) in a system is available because all other possible trajectories are really not accessible. This holds for instance for biological evolution, where the available data concerns only those evolutionary paths that have led to present-time organisms^29^. There, the evolutionary trajectory could be considered as a long time series of events, where the final state is the present^1^,^2^ with the special condition that all other possible “presents” will never be accessible. Indeed, in population genetics it was shown that following the trajectory of fixation of a given allele leads to fictitious selection mechanisms. This is primarily due to conditioning that the given event of fixation happens^29^. In a similar manner, this observation is related to the anthropic hypothesis in cosmology^30^,^31^,^32^. In all these cases, within the metaphor of the drunkard’s walk, the “bar” is the available data, the successful simulation or experiment, the present set of organisms and ecosystems, or the present set of physical laws in our universe.

## Conclusions

When we possess a complete knowledge of the state space of a process, like it is the case in the drunkard’s walk, the strength of the bias can be computed and so the true physical laws of the system can be estimated from observations. However, in systems where a subset of the state space is not accessible, either because one deliberately eliminates some of the realizations of the process (e.g. negative results) or because some parts of the state space are still unknown or completely inaccessible, as it is most of the time the case in complex biological systems, this bias cannot be computed. In this paper, by using well established methods for conditioned processes we have developed a simple analysis to show that the program of finding the microscopic models from observations is doomed to fail. Moreover, we have shown that the agreement between the predictions of the (fictitious) model and the data is another form of circular analysis. As such, circular analysis generically corresponds to a restriction of the state space of the process or at least to a restriction of the field of observation to a certain part of the state space. Nevertheless, a conscious restriction might be sometimes useful or necessary in order to develop a first level of understanding. At the validation level, however, every such conditioning must be suspended in order to reveal the existence of any bias.

The conclusion is, at first sight, disarming: as long as we do not know every state of the universe, the microscopic physics that we can build from macroscopic observations alone is restricted by a Doob’s *h*-transform of unknown strength. However, we cannot really know what the unknown states are until an experimental technique makes them observable and thus accessible. Therefore, inference based solely on historical or global observations might have difficulties in finding the true laws of nature even for those states or phenomena that are supposed to be known. A partial solution is to be aware that there may always be an involuntary selection. In experimental sciences, this means that we should pay a stronger attention to negative results. At the same time, one must continue to develop a theoretical understanding of the microscopic dynamics to predict macroscopic patterns, in order to discover contradictions with the laws of nature as they might be inferred from the observation of macroscopic phenomena.

## Additional Information

**How to cite this article**: Valleriani, A. Circular analysis in complex stochastic systems. *Sci. Rep.* **5**, 17986; doi: 10.1038/srep17986 (2015).

## Supplementary Material

Supplementary Information
