# Supplementary material for: Circular analysis in complex stochastic systems
Source: Sci Rep. 2015 Dec 10;5:17986. doi: 10.1038/srep17986 (PMC4675072; doi:10.1038/srep17986)
Supplement: Supplementary Information [file srep17986-s1.pdf]

Supplementary note

Circular analysis in complex stochastic systems

Angelo Valleriani

Abstract

In these supplementary materials I deal with a derivation and a few additional technical generalizations of the discussion made in the main article. Readers interested only in the concepts do not need to read these materials. The first section is devoted the drunkard's walk. The following sections contain extended material and generalizations to any kind of Markov chain in discrete and continuous time. The level is pedagogical.

S1 The drunkard's walk

We model this process with what is called the one-dimensional random walk in discrete time. The time therefore measures only the number of steps and it will be called epoch or step in this and the next section. The basic definition of this walk is that the walker that is in position i at epoch t, will be either in position $i+1$ or $i-1$ at epoch $t+1$. If it moves to position $i+1$ we say that it has made a step to the right, towards the bar. We will also assume that the probability to jump left or right does not depend on the history of the process but depends only on the most recent position. We will call this the Markov property, even if the definition of this property is actually more general than that. If we think in terms of realizations, we define the stochastic variable X_t, which gives us the position of the walker. Namely, if $X_t = i$ we will say that the walker is at position i at epoch t or after step t. In this and in the following sections I will often follow the same notation as in [1].

The state space of the process is the set of positions that can be taken by the walker. Thus the state space is the set of values that can be taken by the variable X_t. We thus define it with

$$\sigma = \{\mathrm{home}, 1, 2, \ldots, n, \mathrm{bar}\}, \tag{S1}$$

where the states $\{1, 2, \ldots, n\}$ are called transient states and the states $\{\mathrm{home}, \mathrm{bar}\}$ are considered as absorbing states. The reason for considering them as absorbing states is that we consider a trajectory of the walker as finished when it reaches either the bar or home.

Given that the walker is at position i, we define now the rules of the walk by assigning the probability that it jumps left or right. In the manuscript, I have considered the

probabilities p and q to jump right or left from any state, respectively. This was done to streamline the discussion by keeping the technical level as pedagogical as possible but it is by no means necessary. Therefore, I will try here to be minimally more general than that and leave the general case to the next section. The probabilities p and q are thus defined as

$$\begin{array}{rcl} P(i \to i+1) & \equiv & \Pr\{X_{t+1} = i+1 \mid X_t = i\} \; = \; p \\ P(i \to i-1) & \equiv & \Pr\{X_{t+1} = i-1 \mid X_t = i\} \; = \; q, \end{array} \tag{S2}$$

where $p + q = 1$ since we do not allow for any other transition. In (S2) the variable i can take any value on the subspace of the transient states, *i.e.*, $i = 1, 2, \dots n$. The probabilities p and q do not need to be equal but they have been chosen to not depend on i. Thus, to cover the whole state space σ defined in (S1) we need to add the following two additional transition probabilities

$$\begin{array}{rcl} \Pr\{X_{t+1} = \text{home} \mid X_t = \text{home}\} & = & 1 \\ \Pr\{X_{t+1} = \text{bar} \mid X_t = \text{bar}\} & = & 1\,, \end{array} \tag{S3}$$

which is what characterizes home and bar as absorbing states.

A realization of the walk is the set of positions that the walker visits until it reaches either home or the bar. Whatever is the initial or starting point of the walk, the time to reach any one of the two absorbing states is a random variable. We will call this random variable T and define it as follows

$$T_A \; = \; \min\{t \geq 0 \text{ s.t.}\, X_t \in \{\text{home}, \text{bar}\}\}\,, \tag{S4}$$

which means that T_A is the smallest time epoch at which the walker is found either at home or in the bar. For any given realization, T_A is obviously an integer number. Once the process has reached one of the absorbing states it does not leave it anymore until a new walk starts. Since the state space in finite and the walker can reach any state when it starts in one of the transient states, we can be sure that absorption in one of the absorbing states is certain. This means that T_A is certainly finite. This allows us to define the stochastic variable X_∞ as the value taken by the stochastic variable X when we wait a time larger than any finite number. In simpler terms, X_∞ is the state at which we will find the walker if we wait long enough until it has reached either home or the bar.

Since X_∞ is a random variable that can be equal either to bar or to home, we can define the probabilities to take these two values. These probabilities are clearly dependent on the initial condition, namely on the value of X_0, *i.e.*, the value that X had at the beginning of the realization. We have thus

$$\begin{array}{rcl} B_i & = & \Pr\{X_\infty = \text{bar} \mid X_0 = i\} \\ H_i & = & \Pr\{X_\infty = \text{home} \mid X_0 = i\}\,, \end{array} \tag{S5}$$

where i now can take any value in σ. Since absorption is exclusive, we have $B_i + H_i = 1$. Therefore, once we know B_i from all i, we automatically know also H_i. To compute

B_i for any value of i we just need to solve the following set of equations (see [1] for an intuitive explanation)

$$B_i \;=\; pB_{i+1} + qB_{i-1}\,, \tag{S6}$$

taking into account that $B_{\text{home}} = 0$ and $B_{\text{bar}} = 1$. The result is that, for $x = q/p$, where p and q were defined in (S2), we have

$$B_i \;=\; \frac{1 - x^i}{1 - x^{n+1}}\,, \tag{S7}$$

with a limiting value $B_i = i/(n+1)$, as $x \to 1$.

We are now ready for the main equation of the manuscript. When we consider processes that have been conditioned to finish in a given specific absorbing state, we can include this condition in any of the quantities of interest. In particular, we are interested in the transition probabilities

$$\begin{aligned} P^{(\text{bar})}(i \to i+1) \;&\equiv\; \Pr\{X_{t+1} = i+1 \mid X(t) = i\,,\, X_\infty = \text{bar}\} \\ P^{(\text{bar})}(i \to i-1) \;&\equiv\; \Pr\{X_{t+1} = i-1 \mid X(t) = i\,,\, X_\infty = \text{bar}\}\,, \end{aligned} \tag{S8}$$

where the additional condition in the rhs expresses the fact that the probabilities have to be computed only on the set of trajectories ending at bar. Intuitively, it is not obvious that the condition on X_∞ should modify the jump probabilities in position i. We follow now a simple application of the rule that relates the conditional to the joint probabilities, *i.e.*, if A and B are two events, we have

$$\Pr\{A,\, B\} \;=\; \Pr\{A \mid B\}\Pr\{B\}\,. \tag{S9}$$

Let us then consider the first of (S8). We have

$$\begin{aligned} &P^{(\text{bar})}(i \to i+1) \;\equiv\; \Pr\{X_{t+1} = i+1 \mid X(t) = i\,,\, X_\infty = \text{bar}\} \\ &= \frac{\Pr\{X_{t+1} = i+1\,,\, X_t = i\,,\, X_\infty = \text{bar}\}}{\Pr\{X(t) = i\,,\, X_\infty = \text{bar}\}} \\ &= \frac{\Pr\{X_\infty = \text{bar} \mid X(t) = i\,,\, X_{t+1} = i+1\}\Pr\{X(t) = i\,,\, X_{t+1} = i+1\}}{\Pr\{X(t) = i\,,\, X_\infty = \text{bar}\}} \\ &= \frac{\Pr\{X_\infty = \text{bar} \mid X_{t+1} = i+1\}\Pr\{X_{t+1} = i+1 \mid X(t) = i\}\Pr\{X(t) = i\}}{\Pr\{X_\infty = \text{bar} \mid X(t) = i\}\Pr\{X(t) = i\}} \\ &= \frac{B_{i+1}}{B_i}P(i \to i+1) \end{aligned} \tag{S10}$$

which reproduces the equation shown in the main manuscript. For $p = q = 1/2$ this leads to $P^{(\text{bar})}(i \to i+1) = (i+1)/2i$, which is systematically larger than $p = 1/2$. Therefore, the transition probabilities obtained from selected trajectories ending at the bar are apparently different from the true transition probabilities. The condition on the end point of the trajectories introduces a bias. This condition in the future is completely equivalent to looking back into the past from a preselected observational viewpoint.

S1.1 The stationary drunkard's walk

Consider a slightly modified process, in which the walker can visit home and bar several times (or a random number of times) before settling down and stopping. Let us call T the time[1] at which it stops and let us consider only those trajectories for which $X_T = \text{bar}$. Then we have

$$\begin{aligned} &\Pr\{X_{T-t} = i+1 \mid X_{T-t-1} = i,\, X_T = \text{bar}\} = \\ &= \frac{\Pr\{X_t = \text{bar} \mid X_0 = i+1\}}{\Pr\{X_{t+1} = \text{bar} \mid X_0 = i\}} \Pr\{X_{T-t} = i+1 \mid X_{T-t-1} = i\}\,, \end{aligned} \tag{S11}$$

which shows that under the condition $X_T = \text{bar}$, the transition probabilities are time-dependent whereas the true transition probabilities are not. This shows that by selecting the trajectories based on which state is visited last, even if this state was visited several times before and the process is in steady state, the condition biases the transition probabilities and makes them different from the true ones.

S1.2 Classification vs prediction based on patterns

Consider the drunkard's walk with $p = q = 1/2$ based on the definitions given in (S2). Suppose that a trajectory that has finished either at home or at the bar, but we don't know which one of the two, contains, for a given state i a number of transitions from i to $i+1$ larger than the number of transitions from i to $i-1$, i.e.

$$\mathcal{N}(i \to i+1) > \mathcal{N}(i \to i-1)\,. \tag{S12}$$

The inequality (S12) represents thus a simple pattern that one could have found by analyzing many trajectories. We would certainly agree that even when $p = q = 1/2$, this pattern is expected to emerge if the set of trajectories is conditioned to end at the bar, *i.e.*,

$$\Pr\{\mathcal{N}(i \to i+1) > \mathcal{N}(i \to i-1) \mid X_\infty = \text{bar}\} > \Pr\{\mathcal{N}(i \to i+1) > \mathcal{N}(i \to i-1)\}\,. \tag{S13}$$

We can now ask to which extent the discovery of this pattern implies that a trajectory whose end has not been yet revealed has ended more likely at the bar or at home, namely if

$$\Pr\{X_\infty = \text{bar} \mid \mathcal{N}(i \to i+1) > \mathcal{N}(i \to i-1)\} > \Pr\{X_\infty = \text{bar}\}\,, \tag{S14}$$

independently of the initial condition. The answer is obtained by using the Bayes rule on the left hand side, which equals

$$\frac{\Pr\{\mathcal{N}(i \to i+1) > \mathcal{N}(i \to i-1) \mid X_\infty = \text{bar}\}}{\Pr\{\mathcal{N}(i \to i+1) > \mathcal{N}(i \to i-1)\}} \Pr\{X_\infty = \text{bar}\}\,, \tag{S15}$$

which is in general larger than $\Pr\{X_\infty = \text{bar}\}$ because $P_i^{(\text{bar})} > p$.

[1] Here T is an arbitrary time not to be confused with T_A defined in Eq. (S4).

This result could lead to the conclusion that there is a possibility to know in advance, before the end of the trajectory, where the trajectory will end up. This question, however, cannot be answered by this calculation. In fact, what this calculation answers is the following question: given that the pattern (S12) holds and given that the trajectory has already come to an end, which kind of trajectory was it? Where did it end? Since all what has to be predicted has already happened, here we can only classify instead of predicting.

Rather, one should proceed as follows. Take a process starting in state k. Suppose that at time t_2 the process is in state k, *i.e.*, $X_{t_2} = k$ with k different from home or bar. Suppose also that until time t_1, with $0 < t_1 < t_2$, it is known that the event $\mathcal{N}^{(t_1)}(i \to i+1) > \mathcal{N}^{(t_1)}(i \to i-1)$ is true. For simplicity of notation we call this event $\Delta_i(t_1)$. Thus, we ask if it holds that

$$\Pr\{X_\infty = \text{bar} \mid X_{t_2} = k,\, \Delta_i(t_1)\} \;>\; \Pr\{X_\infty = \text{bar} \mid X_{t_2} = k\}\,. \tag{S16}$$

Since $t_1 < t_2$, applying the Markov property shows that the inequality cannot hold and the two terms are equal. As a conclusion, the discovery of patterns within a trajectory does not increase the prediction accuracy beyond the chance level represented by the absorption probabilities.

The most clear technical conclusion to be drawn from these calculations is that a pattern does not mean a rule. If we had ignored the rules used to build the drunkard's walk we would have instinctively taken the pattern given by Eq. (S10) or Eq. (S12) as a characteristic local rule at position i for trajectories ending at the bar. By doing this, we would have sloppily transformed a pattern in a rule. Luckily enough, by construction we know that the rule was $p = q = 1/2$ and that the microscopic dynamics is not affected by the endpoint of the trajectory. The pattern arises therefore not because there are two different rules depending on whether the trajectory ends at home or at the bar. Rather, it is a simple consequence of conditioning the process in its future outcome.

S2 General results for Markov chains in discrete time

I will depart here from the drunkard's walk example and show that the relationship between the conditioned transition probabilities and the true transition probabilities is similar to what has been claimed in the manuscript. I follow here the notation and the definitions of [1] but the most important results of this supplementary information are not contained in that reference.

Let us consider a Markov chain in discrete time on a finite state space. Let the state space be

$$\sigma \;=\; \{0, 1, \ldots, n-1, n, \ldots, N\}\,, \tag{S17}$$

such that $\sigma_0 = \{0, 1, \ldots, n-1\}$ is the subset of the transient states and $\sigma_A = \{n, n+1, \ldots, N\}$ is the subset of the absorbing states. Obviously $\sigma = \sigma_0 \cup \sigma_A$ and the intersection of these two subsets is empty. The cardinality of σ_0 is n and the cardinality of σ_A is $N + 1 - n$. Let X be the state variable so that $X_t = j$ means that the process

is in state j at epoch t. We consider here only time-homogeneous processes, in which the transition probabilities do not depend on time. The one-step transition probability matrix $\mathbf{P}$ is defined as

$$P_{ij} \; = \; \Pr\{X_{t+1} = j \mid X_t = i\} \, , \tag{S18}$$

for $i, j \in \sigma$. The matrix $\mathbf{P}$ has a block structure

$$\mathbf{P} \; = \; \begin{pmatrix} \mathbf{R} & \mathbf{Q} \\ \mathbf{0} & \mathbf{Id} \end{pmatrix} \tag{S19}$$

where $\mathbf{R}$ is a $n \times n$ square matrix that contains the transition probabilities from transient to transient state, the $n \times (N + 1 - n)$ submatrix $\mathbf{Q}$ contains the transition probabilities from the transient to the absorbing states, the $(N+1-n) \times n$ matrix $\mathbf{0}$ contains all zeros and the $(N + 1 - n) \times (N + 1 - n)$ matrix $\mathbf{Id}$ is the unit matrix with zeros everywhere except on the diagonal were it has all ones.

Each realization of the process will eventually end in one of the absorbing states. Whatever is the initial condition on σ_0, the time or number of steps until absorption is an integer random variable. We will call this random variable T_A and define it as follows

$$T_{_A} \; = \; \min\{t \geq 0 \text{ s.t.} \, X_t \in \sigma_A\} \, , \tag{S20}$$

which means that $T_{_A}$ is the earliest epoch at which the process is found in any of the absorbing states. Once the process has reached one of the absorbing states it does not leave it anymore until a new realization starts. Since the state space in finite and the walker can reach any state when it starts in one or the transient states, we can be sure that absorption in σ_A is certain. This means that $T_{_A}$ is certainly finite. This allows us to define the stochastic variable X_∞ as the value taken by the stochastic variable X when we wait a time larger than any finite number. In simpler terms, X_∞ is the state at which we will find the process if we wait long enough until it has reached one of the absorbing states.

The variable X_∞ is thus a random variable in σ_A and its probability mass function on this state space will depend on the initial conditions of the process. The question now is to find out how this probability mass function can be derived by the knowledge of $\mathbf{P}$ defined in (S19). We start by considering the transition probabilities after m steps, defined as

$$P_{ij}^{(m)} \; = \; \Pr\{X_{t+m} = j \mid X_t = i\} \, . \tag{S21}$$

The m-step transition probabilities are given by the m-power of the one-step transition probability matrix $\mathbf{P}$. Thus

$$P_{ij}^{(m)} \; = \; (\mathbf{P}^m)_{ij} \; , \tag{S22}$$

which can be easily computed by considering the block structure of that matrix, as shown in (S19). We have in fact that

$$\mathbf{P}^m \; = \; \begin{pmatrix} \mathbf{R}^m & \left(\sum_{j=0}^{m-1} \mathbf{R}^j\right) \cdot \mathbf{Q} \\ \mathbf{0} & \mathbf{Id} \end{pmatrix} \, , \tag{S23}$$

with the straightforward conclusion that in the limit of large m this matrix should deliver the absorption probabilities, as

$$\lim_{m\to\infty} \mathbf{P}^m = \begin{pmatrix} \mathbf{0} & \mathbf{U} \\ \mathbf{0} & \mathbf{Id} \end{pmatrix}, \tag{S24}$$

where $\mathbf{U}$ is now $n \times (N+1-n)$ matrix that gives the absorption probabilities in any state of σ_A from any transient state of σ_0. The form of this matrix can deduced in different ways [1]. By just taking the limit $m \to \infty$ in (S23) and using the similarity to the geometric series, one obtains

$$\mathbf{U} = (\mathbf{Id} - \mathbf{R})^{-1} \cdot \mathbf{Q}. \tag{S25}$$

In summary, thus, we have

$$U_{ik} = \Pr\{X_\infty = k \mid X_0 = i\}, \tag{S26}$$

with U_{ik} being the matrix element of $\mathbf{U}$ corresponding to the transient state i and to the absorbing state k.

We are now ready to proceed to the general principle of conditioned paths. Let us assume that $k \in \sigma_A$ is a preselected absorbing state and that we consider now only those realizations that end in k. We want to compute the one-step transition probabilities between any two connected transient states under this condition. These are given by

$$P_{ij|k} = \Pr\{X_{t+1} = j \mid X_t = i,\, X_\infty = k\}, \tag{S27}$$

where the condition on k is engraved in the index. By using the probability laws that define the conditional probabilities, and by observing that in the definition of the absorption probability the time at which the initial condition is expressed does not play any role due to the Markov property, we obtain that

$$P_{ij|k} = \frac{U_{jk}}{U_{ik}} P_{ij}, \tag{S28}$$

which shows that the transition probabilities on paths conditioned in their outcome, $P_{ij|k}$, are different from the true or original ones, P_{ij}. The definitions (S27) and (S28) are consistent with the notation used for the conditional probabilities since

$$P_{ij} = \sum_{k\in\sigma_A} P_{ij|k} U_{ik}. \tag{S29}$$

Once the process is conditioned in being absorbed in k, it is equivalent to a Markov chain with just one absorbing state, whose one-step transition probability matrix is different from the "unconditioned" one defined in (S19) and can be formally defined as

$$\mathbf{P}_{|k} = \begin{pmatrix} \mathbf{R}_{|k} & \mathbf{Q}_{|k} \\ \\ \mathbf{0} & \mathbf{1} \end{pmatrix}, \tag{S30}$$

where the subscript $|k$ reminds that this is the matrix derived by conditioning in the future outcome in k. By using Eq. (S28) we can easily derive the relationship between $\mathbf{R}_{|k}$ and $\mathbf{R}$. We just need to define the diagonal matrix

$$\left(\mathbf{U}_{|k}\right)_{ij} = \delta_{ij} U_{jk}\,, \tag{S31}$$

where $i, j \in \sigma_0$ and U_{jk} is defined in (S26). The relationship (S28) then is translated into

$$\mathbf{R}_{|k} = \mathbf{U}_{|k}^{-1} \cdot \mathbf{R} \cdot \mathbf{U}_{|k}\,, \tag{S32}$$

which is a matrix relationship that tells us that all transition probabilities and thus the whole character of the process is affected by conditioning in the final outcome of the process. As a side remark, also the time to absorption is modified by this condition and it is not the same as the time defined in (S20). Indeed, the probability function for T_A can be defined as

$$\Pr\{T_A \leq m \mid X_0 = i\} = \sum_{\ell \in \sigma_A} \Pr\{X_m = \ell \mid X_0 = i\}\,, \tag{S33}$$

whereas for the process conditioned to absorb in k we have

$$\begin{aligned} \Pr\{T_{A|k} \leq m \mid X_0 = i\} &= \textstyle\sum_{\ell \in \sigma_A} \Pr\{X_m = \ell \mid X_0 = i,\, X_\infty = k\} \\ &= \frac{1}{U_{ik}} \Pr\{X_m = k \mid X_0 = i\}\,, \end{aligned} \tag{S34}$$

which is consistent with

$$\Pr\{T_A \leq m \mid X_0 = i\} = \sum_{\ell \in \sigma_A} \Pr\{T_{A|\ell} \leq m \mid X_0 = i\} U_{i\ell}\,. \tag{S35}$$

S2.1 Processes at steady state

One could think that when a process does not have absorbing states and it is at steady state the effect that corrects the transition probabilities disappears. I will show here that this is not the case. We will see that there is an effect of the choice of the viewpoint.

Let us consider, in this subsection only, that we have a Markov chain in discrete time defined solely on the state space $\sigma_0 = \{0, 1, \ldots, n-1\}$. I will assume that all states are recurrent and that all states are communicating. I will also assume that the process is aperiodic so that a unique steady state distribution $\vec{\pi}$ exists. By denoting with $\mathbf{P}$ the $n \times n$ one-step transition probabilities matrix, $\vec{\pi}$ satisfies

$$\vec{\pi} = \vec{\pi} \cdot \mathbf{P}\,. \tag{S36}$$

The elements of $\mathbf{P}$ are defined as usual as

$$P_{ij} \equiv \Pr\{X_{t+1} = j \mid X_t = i\}\,, \tag{S37}$$

and are time independent.

Let us consider now the state $k \in \sigma_0$, such that k is a pre-selected state that we use to make our observations and collect the trajectories. Let us consider now an ensemble of trajectories made of a number of steps T much larger than the characteristic relaxation time of the process, *i.e.*, much larger than $-1/(\log|\lambda|)$ with λ being the largest non-unitary eigenvalue of $\mathbf{P}$. Out of this ensemble we consider the subset made of those trajectories that satisfy $X(T) = k$, namely of the realizations that are in state k at this large time T.

Since the realizations are very long, the memory of the initial condition has been lost. Moreover, during each of the realizations the process will have visited the state k several times, in average $\pi_k T$ times. From this steady state condition, we wish now to evaluate the transition probabilities from state i to state j on the ensemble of trajectories whose only characteristics is to have state k as the last visited state. We do this by substituting t in Eq. (S37) with $T - m$, namely

$$P_{ij|k} \;=\; \Pr\{X_{T-m+1} = j \mid X_{T-m},\, X_T = k\}\,, \tag{S38}$$

where the second condition expresses our restriction to trajectories that satisfy $X_T = k$. By applying Bayes theorem and the Markov property to (S38) we finally obtain

$$P_{ij|k} \;=\; \frac{\left(\mathbf{P}^{m-1}\right)_{jk}}{\left(\mathbf{P}^{m}\right)_{ik}}\, P_{ij}\,, \tag{S39}$$

for $m \geq 1$ and where P_{ij} in the rhs is defined in Eq. (S37). One can readily see that $P_{ij|k}$ is now time-dependent as it varies with m. It is certainly true that the time dependence disappears as m becomes large, *i.e.*, as one goes back in the past of the trajectories, since

$$\lim_{m\to\infty} \left(\mathbf{P}^{m}\right)_{\ell k} \;=\; \pi_k\,, \tag{S40}$$

for any $\ell \in \sigma_0$, by definition of steady state. However, the time dependence that we see in (S39) is unexpected and even if T is very large it may affect the statistics, especially when the sampling is made at small m.

S3 General results for Markov chains in continuous time

In this section I will consider the rates as the fundamental quantities that govern the process and I will discuss how the conditioning on one particular absorbing state, out of many different absorbing state, influences the values of all rates of the process. Some of the results presented here appeared previously in [2, 3, 4]. In particular, in [2] the Doob's h-transform was introduced in applied continuous time Markov chains and was subsequently formalized in [5]. In the context of conditioning on rare events, the Doob's h-transform has been discussed in [6]. In a more general context relating equilibrium and non-equilibrium processes it has been discussed in [7].

Let us consider a Markov chain in continuous time on a finite state space. Let the state space be

$$\sigma = \{0, 1, \ldots, n-1, n, \ldots N\}\,, \tag{S41}$$

such that $\sigma_0 = \{0, 1, \ldots, n-1\}$ is the subset of the transient states and $\sigma_A = \{n, n+1, \ldots, N\}$ is the subset of the absorbing states. Obviously $\sigma = \sigma_0 \cup \sigma_A$ and the intersection of these two subsets is empty. The cardinality of σ_0 is n and the cardinality of σ_A is $N+1-n$. Let X be the state variable so that $X(t) = j$ means that the process is found in state j at time t (in this section the time is a continuous variable and we use the notation $X(t)$ instead of X_t, which was used when time was discrete). We consider here only time-homogeneous processes.

The elementary conditional probabilities that completely define the process is governed by the rates ω_{ij}, which are defined as

$$\Pr\{X(t+\tau) = j \mid X(t) = i\} = \omega_{ij}\tau + o(\tau)\,, \tag{S42}$$

for any $i, j \in \sigma$, as $\tau \to 0$ and where $o(\tau)$ contains terms of order higher than τ in this limit. Let now $\vec{P}(t)$ be the $(N+1)$-dimensional vector that gives the probabilities at time t that the process is in any of the states in σ given the initial condition $\vec{P}(0)$. Formally, an element of $\vec{P}$ under this initial condition is defined as

$$P_i(t) = \Pr\{X(t) = i \mid \vec{P}(0)\}\,, \tag{S43}$$

for any $i \in \sigma$. It follows from (S42) that this probability vector is the solution of the Master equation

$$\frac{\mathrm{d}\vec{P}(t)}{\mathrm{d}t} = \vec{P}(t)\,\mathbf{\Omega}\,, \tag{S44}$$

where $\mathbf{\Omega}$ is the matrix whose element (i, j) is the rate ω_{ij}. The matrix $\mathbf{\Omega}$ is also called the infinitesimal generator of the process [1]. This name comes from the fact that the solution of the Master equation an be formally written as

$$\vec{P}(t) = \vec{P}(0)\exp\left(\mathbf{\Omega}t\right)\,, \tag{S45}$$

where the diagonal elements ω_i are fixed such that $\sum_j \omega_{ij} = 0$. Note, as a side remark, that there are two ways to discretize this process. One way is to create the embedded process in discrete time by substituting all exponentially distributed dwell times with times identical to one time unit and by defining the jump probabilities $P_{ij} = \omega_{ij}/\sum_k \omega_{ik}$, for any $i \neq j$. Another way is to consider (S45) at time intervals of length τ. In matrix form this gives the one-step transition probability matrix

$$\mathbf{P} = \exp\left(\mathbf{\Omega}\tau\right)\,, \tag{S46}$$

where one step has duration of τ time units. In this form, the probabilities to find the process in any state after $n\tau$ time units is just given by $\mathbf{P}^n$. If there is any technical reason that favors working with the discretized version (S46), then the results of the previous section hold. For more elementary properties of this kind of processes the reader

should consult [1]. The discretization defined in (S46) is commonly used in studies where molecular dynamics (MD) simulations are transformed in discrete time Markov chains (see [8] and references therein).

The structure of the state space, where we have labeled the transient and the absorbing states by keeping them separated, allows to write the solution (S45) explicitly for the transient and absorbing states. This is due to the fact that the infinitesimal generator has a block form

$$\mathbf{\Omega} = \begin{pmatrix} \mathbf{S} & \mathbf{A} \\ \mathbf{0} & \mathbf{0} \end{pmatrix}, \tag{S47}$$

where $\mathbf{S}$ is a $n \times n$ matrix containing the transition rates between the transient states, $\mathbf{A}$ is a $n \times (N+1-n)$ matrix containing the transition rates between the transient and the absorbing states. This matrix structure leads to the very simple result of matrix power

$$\mathbf{\Omega}^m = \begin{pmatrix} \mathbf{S}^m & \mathbf{S}^{m-1}\mathbf{A} \\ \mathbf{0} & \mathbf{0} \end{pmatrix}, \tag{S48}$$

which turns out to be very useful in (S45). Indeed, if we split $\vec{P}$ in a vector that refers only to the transient states and a vector that refers only to the absorbing states, *i.e.*, $\vec{P}(t) = \{\vec{P}_0(t), \vec{P}_A(t)\}$, and we assume that the initial condition is only on the transient states, *i.e.*, $\vec{P}(0) = \{\vec{P}_0(0), 0, \ldots, 0\}$, we obtain

$$\begin{aligned} \vec{P}_0(t) &= \vec{P}_0(0)\left[\exp\left(\mathbf{S}t\right)\right] \\ \vec{P}_A(t) &= \vec{P}_0(0)\left[\mathbf{S}^{-1}\left[\exp\left(\mathbf{S}t\right) - \mathbf{Id}\right]\mathbf{A}\right], \end{aligned} \tag{S49}$$

which have the obvious limits

$$\begin{aligned} \lim_{t\to\infty} \vec{P}_0(t) &= 0 \\ \lim_{t\to\infty} \vec{P}_A(t) &= \vec{P}_0(0)\left[-\mathbf{S}^{-1}\mathbf{A}\right] \equiv \vec{P}_0(0)\,\mathbf{U}. \end{aligned} \tag{S50}$$

The matrix $\mathbf{U}$ defined in this limit is a $n \times (N+1-n)$ matrix that contains the absorption probabilities from any of the transient states (rows) to any of the absorbing states (columns). More formally, let us define the random variable T_A as the absorption time, namely as the time until the process has reached any of the absorbing states

$$T_A = \inf\{t \geq 0 \text{ s.t. } X(t) \in \sigma_A\}, \tag{S51}$$

and we define $X_\infty \in \sigma_A$ as the value that the stochastic variable $X(t)$ has taken at the time point in which absorption takes place. Thus, the element U_{ik} of the matrix $\mathbf{U}$ is defined as

$$U_{ik} = \Pr\{X_\infty = k \mid X(0) = i\}. \tag{S52}$$

As Eq. (S50) shows, the knowledge of the matrices $\mathbf{S}$ and $\mathbf{A}$ is sufficient to compute the matrix $\mathbf{U}$ by using matrix algebra alone. Furthermore, the statistics of the absorption time T_A can be computed from the solution (S49) by

$$\Pr\{T_A \leq t \mid \vec{P}(0)\} \;=\; \vec{P}_A(t) \cdot 1_A^\top \,, \tag{S53}$$

where $1_A^\top$ is a column vector of length $N+1-n$ all made of 1s so that the rhs of this formula is just the sum of the components of the vector $\vec{P}_A(t)$. From the second of (S49) one obtains the probability density $\phi_A(t)$ for the absorption time T_A as

$$\phi_A(t) \;=\; \vec{P}_0(0)\,[\exp(\mathbf{S}t)\,\mathbf{A}] \cdot 1_A^\top \,, \tag{S54}$$

from which one derives the expectation value of T_A as

$$\mathrm{E}[T_A \mid \vec{P}(0)] \;=\; -\vec{P}_0(0)\left[\mathbf{S}^{-1}\mathbf{U}\right] \cdot 1_A^\top \,, \tag{S55}$$

by employing only matrix algebra.

S3.1 Processes conditioned to be absorbed in one out of many absorbing state

After the preliminaries, let us thus consider one given selected absorbing state $k \in \sigma_A$ and let us consider only those trajectories that start in $\vec{P}_0(0)$ and are absorbed in k. All these trajectories thus describe a Markov chain with one single absorbing state. By employing the same notation as before, we will consider a process whose infinitesimal generator is the matrix $\mathbf{\Omega}_{|k}$ conditioned that the realizations are absorbed in k. The elements $\omega_{ij|k}$ of the matrix $\mathbf{\Omega}_{|k}$ will have a relationship with the elements ω_{ij} of the unconditioned process. Indeed, $\omega_{ij|k}$ is defined as

$$\Pr\{X(t+\tau) = j \mid X(t) = i,\, X_\infty = k\} \;=\; \omega_{ij|k}\tau \,+\, o(\tau)\,, \tag{S56}$$

for $i, j \in \sigma_0$ when $\tau \to 0$. The lhs of this definition, however, can be transformed using Bayes theorem as follows

$$\begin{aligned}&\Pr\{X(t+\tau) = j \mid X(t) = i,\, X_\infty = k\} \;=\\ &=\; \frac{\Pr\{X_\infty = k \mid X(0) = j\}}{\Pr\{X_\infty = k \mid X(0) = i\}}\Pr\{X(t+\tau) = j \mid X(t) = i\}\,,\end{aligned} \tag{S57}$$

which upon using (S56), (S52) and (S42) leads to

$$\omega_{ij|k} \;=\; \frac{U_{jk}}{U_{ik}}\omega_{ij}\,, \tag{S58}$$

for $i, j \in \sigma_0 \cup \{k\}$. This equation shows that the observed rates on processes conditioned to be observed only from state k are different from the true rates.

We can formalize this result to achieve a full description of the conditioned chain as follows. Let us start by introducing the $n \times n$ diagonal matrix $\mathbf{U}_{|k}$ that contains on the diagonal only the absorption probabilities from each transient state to the state k

$$\left(\mathbf{U}_{|k}\right)_{ij} = \delta_{ij} U_{jk} , \tag{S59}$$

where the probabilities U_{jk} have been defined in (S52). Then, based on (S58) we define the submatrix $\mathbf{S}_{|k}$ related to the submatrix $\mathbf{S}$ defined in (S48) as

$$\mathbf{S}_{|k} = \mathbf{U}_{|k}^{-1} \cdot \mathbf{S} \cdot \mathbf{U}_{|k} , \tag{S60}$$

where one should make the important observation that the diagonal elements of $\mathbf{S}_{|k}$ are the same as the diagonal elements of the matrix $\mathbf{S}$, thus ensuring that the dwell time distributions are not affected by the conditioning. In complete analogy, we can also define the $n \times 1$ matrix

$$\left(\mathbf{A}_{|k}\right)_{i} = \omega_{ik}/U_{ik} , \tag{S61}$$

and finally the $(n+1) \times (n+1)$ infinitesimal generator for the conditioned process to be absorbed in k is

$$\mathbf{\Omega}_{|k} = \begin{pmatrix} \mathbf{S}_{|k} & \mathbf{A}_{|k} \\ 0 & 0 \end{pmatrix} , \tag{S62}$$

from which we can solve the corresponding Master equation as we have done earlier with the unconditioned process. An important test that $\mathbf{\Omega}_{|k}$ is a true infinitesimal generator is that the sum over all column of each row is zero. This is achieved by observing that in the process conditioned to absorb in k, the probability of absorption in k is one. Therefore

$$-\mathbf{S}_{|k}^{-1} \cdot \mathbf{A}_{|k} = 1^{\top} , \tag{S63}$$

from which, after multiplying left and right by $\mathbf{S}_{|k}$, we have

$$\mathbf{S}_{|k} \cdot 1^{\top} = -\mathbf{A}_{|k} . \tag{S64}$$

This demonstrates that the matrix $\mathbf{\Omega}_{|k}$ is a true infinitesimal generator of the process conditioned to absorb in k. Furthermore, the fact that the diagonal elements are unchanged demonstrates that the dwell times on each of the single transient states are unchanged, *i.e.*, are distributed as in the non-conditioned chain. This is a counter-intuitive but quite interesting property: the dwell times are independent of the knowledge that we may have on the full state space of the process. Yet, the determination of the rates from trajectories ending in state k would reveal values of the rates that are not the true values.

S3.2 Processes at steady state

We can consider here a time homogeneous, continuous time Markov chain on the state space $\sigma_0 = \{0, 1, \ldots, n-1\}$ made of recurrent states. We assume that this process is

governed by the $n \times n$ infinitesimal generator $\mathbf{\Omega}$ containing all the rates. We assume that the process attains a unique steady state $\vec{\pi}$, which is the solution of

$$\vec{\pi} \cdot \mathbf{\Omega} \,=\, 0\,, \tag{S65}$$

and is the limiting behavior as $t \to \infty$ of the solution

$$\vec{P}(t) \,=\, \vec{P}(0)\, \exp\left(\mathbf{\Omega} t\right)\,, \tag{S66}$$

for any initial condition $\vec{P}(0)$.

Consider the largest (negative) eigenvalue λ of $\mathbf{\Omega}$ and take the inverse of its absolute value $1/|\lambda|$ as the most relevant time scale in the system. We consider then realizations of duration $t_s \gg 1/|\lambda|$ and select those that satisfy $X(t) = k$, for $k \in \sigma_0$ where t can be different for each individual trajectory but satisfies $t > t_s$. We then time-lock these trajectories by shifting each individual t to a common value T, such that all trajectory now satisfy $X(T) = k$. Here, T is an arbitrary time whose specific value is irrelevant for what follows. We find that

$$\begin{aligned} &\Pr\{X(T-t+\tau) = j \mid X(T-t) = i,\, X(T) = k\} \,=\\ &= \frac{\Pr\{X(t-\tau) = k \mid X(0) = j\}}{\Pr\{X(t) = k \mid X(0) = i\}} \Pr\{X(u+\tau) = j \mid X(u) = i\} \end{aligned} \tag{S67}$$

from which, upon using the definition of rate and by taking the limit $\tau \to 0$, we obtain

$$\omega_{ij|k}(t) \,=\, \frac{P_{jk}(t)}{P_{ik}(t)} \omega_{ij}\,, \tag{S68}$$

where $P_{ik}(t)$ and $P_{jk}(t)$ are the component k of the solution vector $\vec{P}(t)$ given in Eq. (S66) when the initial condition is centered in state i and j, respectively. Eq. (S68) tells us that even at steady state the sampling introduces a bias in the value of the rates, especially at short time scales before the end of the trajectory. This bias makes the rates apparently dependent on time. Under this point of view, short steady state trajectories all finishing in the same state lead to misleading values of the rates.

References

[1] H. M. Taylor, S. Karlin, *An Introduction to Stochastic Modeling, Third Edition* (Academic Press, 1998).

[2] A. Valleriani, S. Liepelt, R. Lipowsky, *EPL* **82**, 28011 (2008).

[3] P. Keller, A. Valleriani, *J Chem Phys* **137**, 084106 (2012).

[4] P. Keller, S. Roelly, A. Valleriani, *Methodology and Computing in Applied Probability* (2013).

[5] P. Keller, S. Roelly, A. Valleriani, *Stochastic Models* **31**, 98 (2015).

[6] R. Chetrite, H. Touchette, *Annales Henri Poincaré* pp. 1–53 (2014).

[7] R. M. L. Evans, *Phys Rev Lett* **92**, 150601 (2004).

[8] G. R. Bowman, V. Pande, F. Noé, eds., *An Introduction to Markov State Models and Their Application to Long Timescale Molecular Simulation*, Advances in Experimental Medicine and Biology, 797 (Springer, 2014).
